# Integrating greenhouse gas capture and C1 biotechnology: a key challenge for circular economy

**DOI:** 10.1111/1751-7915.13991

**Published:** 2021-12-14

**Authors:** José L. García, Beatriz Galán

**Affiliations:** ^1^ Environmental Biotechnology Laboratory Department of Microbial and Plant Biotechnology Centro de Investigaciones Biológicas Margarita Salas (CIB‐MS, CSIC) Madrid Spain

## Abstract

State of the art on the valorisation of C1 carbon sources obtained either from natural or anthropogenic origins as a key challenge for the circular economy.

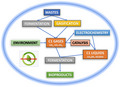

## Introduction

Life is supported by a small number of elements of the periodic table, i.e. between 25 and 28 elements according to different criteria. While all of them are relevant, carbon is the key element that explains life in our planet, even though elemental carbon cannot be used directly as a carbon source by any organism. Carbon must be previously bound to other elements, forming small or very large molecules to become metabolizable by the living beings. In fact, life arose from the combination of small molecules such as hydrogen (H_2_), water (H_2_O), ammonia (NH_3_), hydrogen sulfide (H_2_S), carbon monoxide (CO), carbon dioxide (CO_2_), methane (CH_4_), formaldehyde (H_2_CO) or hydrocyanic acid (HCN), within others. Several of them contain a single carbon atom and form part of a group of molecules named as C1 compounds. Interestingly, all these prebiotic small molecules still remain in the biosphere, and some of them are quite abundant and useful to support life, remaining many micro‐ and macroorganisms able to use them as carbon and/or energy sources. The aim of this editorial is to analyse the present and future prospects and the valorization of C1 carbon sources obtained either from natural or anthropogenic origin through microbial biotechnology as a key challenge for the ‘Green Deal’ and the circular economy.

To focus this analysis, it is important to define the scope of C1 compounds. Although C1 compounds are usually defined as substances that contains a single carbon atom, some authors extend this scope to those compounds that contain carbon atoms without C‐C bonds. Among the first, we can consider CO, CO_2_, CH_4_, H_2_CO, methanol (CH_3_OH), formic acid (HCOOH), methylamine (CH_3_NH_2_), methanethiol (CH_3_SH), different halomethanes (e.g. CHCl_3_) and others. Among the latter, we can list, for instance, dimethyl or trimethyl amines, dimethylsufides, dimethylsulfoxide, dimethylsulfone, dimethylformamide and others. Special mention should be made of soluble or insoluble mineral carbonates that can be converted into CO_2_ under specific environmental conditions and then used by some organisms as a carbon source (Kral *et al*., 2014).

On the other hand, we should consider that not all C1 compounds are equally applicable as potential feedstock for industrial scale biomanufacturing mainly due to their limited availability. The most relevant C1 compounds for biotech purposes include CO_2_, CO, CH_4_, HCOOH and CH_3_OH. Significantly, CO_2_ and CH_4_ are greenhouse gases that are increasing their concentration in the atmosphere due to the large current anthropogenic activity, and consequently, developing biotechnology processes aimed at their sequestration and transformation is essential for planet survival. Additionally, other anthropogenic gases such as power plant flue gas, steel mill gas, anaerobic digestion‐derived biogas, synthesis gas (syngas) and others produced by gasification of organic waste are abundant, rich in C1 substrates (i.e. CO, CO_2_, CH_4_) and, therefore, useful to develop different bioprocesses. Finally, HCOOH or CH_3_OH derived from catalytic processing of CO_2_ or from other sources are liquid substances more amenable than C1 gases to transportation and more affordable for a microbial utilization, due to their higher water solubility.

Except CO_2,_ the other relevant C1 compounds mentioned above can be used both as carbon and energy sources. Therefore, metabolizing CO_2_ requires an additional energy source that can be provided by light, H_2_, CO, electricity or some organic and inorganic compounds. Although some of these C1 compounds can be metabolized by plants and animals, this analysis will be focussed only in the biotech processes based on C1‐utilizing microbes including bacteria, fungi, microalgae and archaea. Native and synthetic C1 assimilation pathways have been used to validate the transformation of C1 compounds to biofuels, and biobased chemicals or even to food and feed (single cell protein) as industrially promising manufacturing procedures, but a deeper understanding of the governing mechanisms of C1 metabolic pathways is needed to develop most efficient C1‐based biotech processes (Jiang *et al*., 2021).

**Fig. 1:**
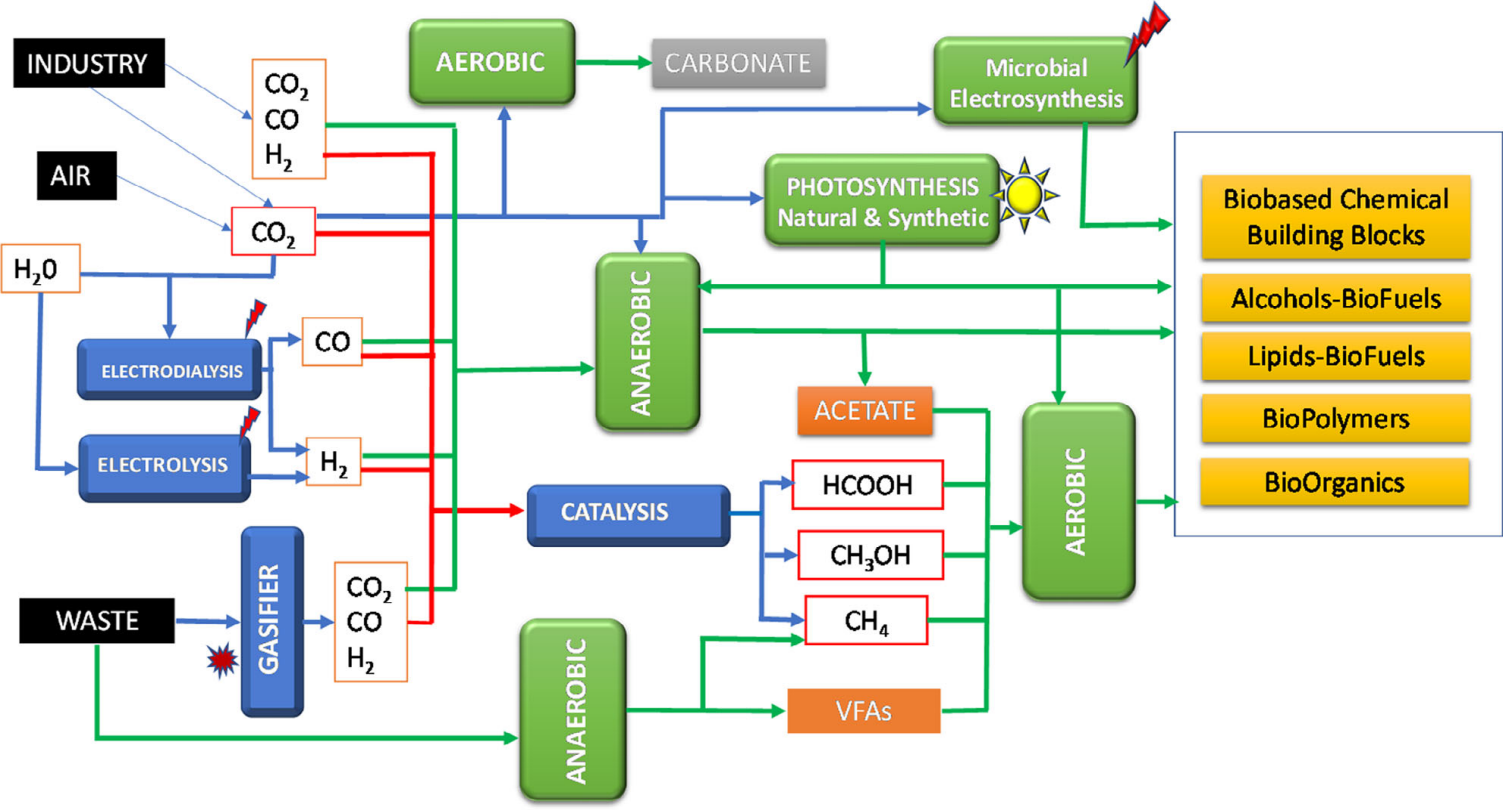
Main biomanufacturing processes that can be carried out using C1 compounds.

Finally, we have to consider that a circular bioeconomy based on C1 compounds has the potential to sustainably produce a large number of compounds, at the same time that can contribute to reduce accumulation of C1 greenhouse and waste gases responsible of climate change, such as CO_2_ and CH_4_. Moreover, technologies that facilitate treatments of all kind of organic waste by gasification followed by carbon capture and conversion of gases into useful products will help also to mitigate climate change by enabling a circular carbon economy (Fackler *et al*., 2021a; Wood *et al*., 2021). Figure 1 shows a scheme of the most relevant biomanufacturing processes that can be carried out using C1 compounds and that will be briefly reviewed hereinafter.

## Fermentations of C1 gases

### Syngas fermentation

We currently have at our disposal a collection of more than 100 isolated anaerobic bacteria named acetogens that synthesize acetyl‐CoA from CO or from CO_2_ plus H_2_ (Bengelsdorf *et al*., 2016; Takors *et al*., 2018; Müller, 2019; Jin *et al*., 2020; Katsyv and Müller, 2020; Lemaire *et al*., 2020; Bourgade *et al*., 2021). These organisms use CO and CO_2_ as substrates for the methyl or carbonyl branches of the Wood–Ljungdahl pathway that produce acetyl‐CoA as metabolic precursor. Acetogens can grow using syngas that is mainly composed of CO_2_, CO and H_2_, a mixture of CO_2_ and H_2_ or only CO. These microorganisms have been used to produce acetate, ethanol, 2,3‐butanediol or butyrate as the most relevant products although others such as acetone or butanol can also be produced from syngas by genetic modifications (Minton *et al*., 2016; Jin *et al*., 2020; Bourgade *et al*., 2021). In this sense, several industrially useful acetogenic bacteria have been already modified using synthetic biology tools, such as *Clostridium ljungdahlii* (Köpke *et al*., 2010; Molitor *et al*., 2016; Zhang *et al*., 2020a), *Clostridium autoethanogenum* (Liew *et al*., 2016; Fackler *et al*., 2021b), *Acetobacterium woodii* (Straub *et al*., 2014) or *Moorella thermoacetica* (Kita *et al*., 2013; Kato *et al*., 2021).

Acetogenic bacteria can be used not only to capture CO_2_ or CO produced as contaminants by anthropogenic activities, but also as a biological alternative to transform syngas into valuable products within the gasification stream of a chemical refinery. To reduce the volume of organic waste (or biomass) generated in cities or industries, it is possible to construct biorefineries that transform organic waste into syngas through different gasification processes (Chan *et al*., 2021; Fackler *et al*., 2021b). Up to now, syngas has been further transformed into different chemicals and fuels of industrial interest by using Fischer–Tropsch catalytic procedures. However, more recently, several research approaches from the academia and the industry (e.g. LanzaTech, IneosBio, Coskata) have demonstrated that syngas can be alternatively transformed into valuable products by using bacterial syngas fermentations (Molitor *et al*., 2017; Bengelsdorf *et al*., 2018; De Tissera *et al*., 2019). However, the efficiency of syngas fermentation is still low and needs to be improved to compete with chemical catalysis (Phillips *et al*., 2017; Sun *et al*., 2019; Geinitz *et al*., 2020).

### CO metabolism

CO, one component of syngas, is a highly toxic compound for most living beings, but there are many microbes which can deal with its toxicity and use it as a carbon and energy source (Robb and Techtmann, 2018; Cordero *et al*., 2019; Duan *et al*., 2021). Curiously, CO occurs at relatively high concentration in Mars’ atmosphere, and it represents a focal point for astrobiological research (King, 2015). CO oxidation coupled to the generation of energy for growth is achieved by aerobic and anaerobic bacteria, and archaea, belonging to the physiological groups of aerobic carboxydotrophic, facultatively anaerobic phototrophic, and anaerobic acetogenic, methanogenic or sulfate‐reducing bacteria. However, not all microbes that metabolize CO are able to grow only using 100% CO. Within the aerobic CO‐oxidizing microorganisms, we can categorize two major groups, the carboxydotrophs and the carboxydovores (Cordero *et al*., 2019). While carboxydotrophs grow chemolithoautotrophically with CO as the sole energy and carbon source when present at elevated concentrations, carboxydovores represent a broader group of bacteria and archaea which oxidize CO at low concentrations, and in contrast to carboxydotrophs require organic carbon to grow. The possibility that CO can be used by some bacteria to convert it into a variety of chemicals and to generate bio‐H_2_ is also promoting a new field of research (Revelles *et al*., 2016, 2017; Robb and Techtmann, 2018; Rodríguez *et al*., 2021).

### CO_2_ capture

Since CO_2_ cannot be used as the sole carbon and energy source, all organisms that capture CO_2_ require an additional source of energy (Claassens *et al*., 2016; Claassens, 2017; Hu *et al*., 2018; Kumar *et al*., 2018; Liang *et al*., 2020). In the case of acetogenic bacteria, CO_2_ is captured by reduction using the energy provided by CO or H_2_. Nevertheless, some acetogenic bacteria can also use electric energy from a cathode to fix CO_2_ by a process called microbial electrosynthesis (MES) (Dessì *et al*., 2021) (see below).

On the other hand, chemolithoautotrophic bacteria can use H_2_ or inorganic compounds as electron donors for energy requirement and growth using CO_2_ as a carbon source. One example of such bacteria is *Cupriavidus necator* that is able to grow and produce different industrial products using CO_2_ and H_2_ under aerobic conditions (Li *et al*., 2020; Nangle *et al*., 2020; Panich *et al*., 2021). Methanotrophs can also sequester CO_2_ and transform it into CH_3_OH using H_2_ or an organic compound as energy source. Therefore, methanotrophs are used as cell factories for the production of a wide range of high‐value products (Sahoo *et al*., 2021).

In addition to H_2,_ there are other compounds used by microbes as electron donors (Gargaud, 2011). In this sense, the most common sulfur compounds utilized as electron donors by denitrifying bacteria (e.g. *Thiobacillus*, *Thiomicrospira*) are hydrogen sulfide (H_2_S), elemental sulfur (S^0^), sulfite (SO_3_^−2^) and thiosulfate (S_2_O_3_^2−^). The aerobic oxidation of ferrous iron (Fe^2+^) to ferric iron (Fe^3+^) is an energy‐yielding reaction, used by some prokaryotes to conserve energy (e.g. *Ferroglobus*). The most common nitrogen compounds used as electron donors for energy conservation are NH_3_ (e.g. *Nitrosomonas*, *Nitrosospira*, *Nitrosococcus* and *Nitrosolubus*) and nitrite (NO^2−^) (e.g. *Nitrobacter*, *Nitrospira* and *Nitrococcus*). A special case of nitrogen‐oxidizing microorganisms corresponds to those capable of carrying out the anoxic oxidation of NH_3_, a process known as anamox. In this case, the electron acceptor is NO^2−^, and the product of the metabolic reaction in addition to proton motive force is the generation of N_2_. This metabolic reaction is carried out by a special type of microorganisms belonging to the Planctomycetes phylum of bacteria.

Phototrophic bacteria utilize light as energy source to capture CO_2_ (Choi *et al*., 2019; Naduthodi *et al*., 2021). It is well known that oxygenic phototrophic cyanobacteria as well as the eukaryotic microalgae, algae and plants use CO_2_ as a carbon source and many reviews have been devoted to show the utility of these bacteria for biotechnological purposes (Singh *et al*., 2018; Veaudor *et al*., 2020; Leong *et al*., 2021; Sarma *et al*., 2021). In addition, anoxygenic phototrophic bacteria can also use CO_2_ to grow and have been utilized for biotech purposes (George *et al*., 2020).

Geologic sequestration of CO_2_, i.e. carbon capture and storage (CCS), is one strategy to reduce the emission of greenhouse gases. Mineralization of CO_2_ into CaCO_3_ is possible if the equilibrium of the reaction of Ca^2+^ with CO_3_^2−^ is moved to the formation of CaCO_3_ under a saturation state. This is achieved in the presence of sufficient dissolved Ca^2+^ at alkaline pH and in the presence of a nucleation substrate. Microbes have been shown to enhance CaCO_3_ precipitation (microbiologically induced calcium carbonate precipitation, MICP) via cation adsorption to negatively charged functional groups on microbe surfaces and by metabolically driven changes in the solution chemistry, which increase mineral saturation and induce nucleation (Castro‐Alonso *et al*., 2019). In general, two metabolic pathways are involved in this biomineralization, i.e. the autotrophic and the heterotrophic pathways (Görgen *et al*., 2020). Autotrophic precipitation of carbonates includes oxygenic and anoxygenic photosynthesis, and non‐methylotrophic methanogenesis. In the heterotrophic pathway, two processes are reported involving sulfur and nitrogen cycles respectively. The microbial induced carbonate precipitation has been biotechnologically used for biocementation of materials (Reddy and Sumit, 2018). Moreover, MICP was investigated for crack repair and the surface treatment of various types of construction materials (Joshi *et al*., 2017; Lee and Park, 2018; Seifan and Berenjian, 2018). Fungi and bacteria can be used in these processes (Menon *et al*., 2019).

However, most interestingly, a number of researches are currently focussed on the creation of new synthetic organisms able to capture CO_2_ using HCOOH or light, opening new frontiers in this field (Woo, 2017; François *et al*., 2020; Liang *et al*., 2020; Satanowski and Bar‐Even, 2020; Satanowski *et al*., 2020). Rewiring *Escherichia coli* for CO_2_ fixation to convert it into sugar may enable diverse biotechnological applications (Antonovsky *et al*., 2017; Flamholz *et al*., 2020). An *E*. *coli* recombinant strain was created to use CO_2_ and HCOOH, and although it still required glucose to grow, authors anticipated that with some additional modifications, it could grow only using CO_2_ and HCOOH (Bang and Lee, 2018; Bang *et al*., 2021). A similar approach had been also carried out using a different metabolic strategy to capture CO_2_ in combination with a complex organic energy source (e.g. glycerol and xylose) (Antonovsky *et al*., 2016; Kerfeld, 2016). Interestingly, the hypothesis was demonstrated, and very recently, a new *E*. *coli* autotrophic recombinant was constructed by laboratory evolution able to capture CO_2_ using HCOOH as the only source of energy (Gleizer *et al*., 2019).

On the other hand, light‐driven CO_2_ sequestration has been achieved in *E. coli* by using self‐assembled cadmium sulfide nanoparticles (Hu *et al*., 2021). Biohybrids had been also investigated in other organisms (Nichols *et al*., 2015; Zhang and Tremblay, 2017; Guo *et al*., 2018; Ding *et al*., 2019; Dogutan and Nocera, 2019; Kumar *et al*., 2019; Sahoo *et al*., 2020) (see below).

Finally, as a proof of concept, a complex *in vitro* system with 17 enzymes was generated to sequester CO_2_ (Schwander *et al*., 2016). An example of how protein engineering and synthetic biology could assist in this mission is the new‐to‐nature glycolyl‐CoA carboxylase created by combining rational design, high‐throughput microfluidics and microplate screens that improved its catalytic efficiency by three orders of magnitude to match the properties of natural CO_2_‐fixing enzymes (Scheffen *et al*., 2021). Moreover, enzymes can also be used in combination with electrochemistry for CO_2_ capture (see below).

### Methane fermentation

CH_4_ can be obtained from natural sources, such as wetlands or animal digestion, along with many anthropogenic activities such as the use of anaerobic digesters (methanogens and biogas) or by thermogenic processes. However, the largest reservoir of CH_4_ is under the seafloor in the form of CH_4_ clathrates. Natural gas is approximately 90% CH_4_. Therefore, it is normal to found many organisms capable of oxidizing CH_4_ in the biosphere that are known as methanotrophs utilizing CH_4_ as the source of carbon and energy. All aerobic methanotrophs oxidize CH_4_ to CO_2_ through a common enzymatic cascade. This oxidation process produces CH_3_OH, CH_2_O and HCOOH as reaction intermediates. Methanotrophs are therefore excellent candidates for CH_4_ sequestration (Sahoo *et al*., 2021). These capabilities enable them as cell factories for a wide range of high‐value products (Nguyen *et al*., 2021). In this sense, methanotrophs have been used to synthesize polyhydroxyalkanoates for plastic sector, single cell proteins for feeding animals and lipids for biofuel production (Wang *et al*., 2020).

## Fermentation of C1 liquids

Common challenges associated with C1 gas fermentation systems are gas‐to‐liquid mass transfer limitations and lower solubility of the gaseous substrates. This problem does not exist when using C1 liquids, such as CH_3_OH or HCOOH. Therefore, CH_3_OH is considered a promising C1 feedstock adding its great availability from different sources (Pirola *et al*., 2018; Simon Araya *et al*., 2020). However, CH_3_OH can inhibit the growth of microorganisms under aerobic conditions, mainly because of the high reactivity of its toxic downstream metabolite H_2_CO. Methylotrophs, including bacteria, such as *Bacillus methanolicus,* and yeasts, such as *Pichia* pastoris, can use CH_3_OH as a carbon and energy source. With some exceptions such as *P. pastoris*, the use of native methylotrophic microorganisms suffers from the drawbacks of poor genetic availability and low metabolic yield, and therefore, engineering non‐native methylotrophic microbes has been used to convert methanol into value‐added products (Zhang *et al*., 2019; Zhan *et al*., 2021). Different bioengineering efforts have shown that these recombinant organisms can be engineered to convert CH_3_OH into biofuels and other commodity chemicals (Bennett *et al*., 2018; Chistoserdova, 2018; Antoniewicz, 2019; Zhu *et al*., 2020). Engineering CH_3_OH metabolic pathways have been mainly carried out in *E. coli*, *Saccharomyces cerevisiae* and *Corynebacterium glutamicum*. However, to date, none of engineered strains can grow on CH_3_OH as the sole carbon source.

On the other hand, HCOOH can be efficiently produced via electrochemical or photochemical catalytic conversion of CO_2_, and it can be directly used as an organic carbon source by microorganisms (Yishai *et al*., 2016; Cotton *et al*., 2020). HCOOH has recently been suggested as an industrial feedstock, although bio‐production based on this carbon source is still not commercially mature (Satanowski and Bar‐Even, 2020). Consequently, the construction of efficient HCOOH‐assimilation pathways in microorganisms is essential for the utilization of cheap, renewable C1 compounds (Mao *et al*., 2020; Tuyishime and Sinumvayo, 2020; Bang *et al*., 2021). Natural microorganisms that possess HCOOH utilization pathways mainly use two strategies to grow on HCOOH as the sole carbon source. In the first one, HCOOH is completely oxidized to generate CO_2_ and reducing equivalents, being Calvin–Benson–Bassham (CBB) cycle an example of this type. In the second one, not all HCOOH is oxidized into CO_2_, while some is directly assimilated via the central metabolism. CBB cycle (reductive pentose‐phosphate cycle) discovered in *C. necator* is the only natural pathway for autotrophic growth on HCOOH, and thus, initial metabolic engineering of HCOOH utilization was mainly concentrated in this pathway. However, new synthetic alternative HCOOH utilization pathways have been recently investigated (Claassens *et al*., 2020; Mao *et al*., 2020). Currently, only some engineered strains of *E. coli* have been able to grow on HCOOH as the sole carbon source although the low cell density and specific growth rate need further improvement (Yishai *et al*., 2018; Bang *et al*., 2020; Kim *et al*., 2020).

## Electrocatalysis

As remarked above, MES is emerging as a promising technology to improve the microbial utilization of C1 compounds (Chu *et al*., 2020; Dessì *et al*., 2021). The first proof‐of‐concept experiment of MES was conducted in 2010 showing that homoacetogens can produce extracellular acetate and 2‐oxobutyrate from CO_2_ with electrons delivered from a graphite electrode (Nevin *et al*., 2010). Since then, many hybrid electro‐biochemical systems have been developed (Li *et al*., 2012; Hwang *et al*., 2015; Bajracharya *et al*., 2017; Gimkiewicz *et al*., 2017; Jang *et al*., 2018; Le *et al*., 2018; Tashiro *et al*., 2018; Yuan *et al*., 2019; Hegner *et al*., 2020).

Nevertheless, electric energy can be also used to synthesize C1 by chemical catalysis and upgraded via microbial fermentation to produce biobased chemicals. In this sense, electrocatalysis represents an attractive strategy with a huge potential in the field of biomanufacturing. The high efficiencies and rates of electrochemical catalysis can be combined with the high selectivity and access to complex end products of microbial catalysis. The electrochemical CO_2_ reduction renders HCOOH or CO that, as stated above, can be used as carbon and energy sources from many microorganisms (Jin *et al*., 2021; Park *et al*., 2021). On the other hand, H_2_ can be generated by electrolysis of H_2_O and used as an energy source to grow. Moreover, the co‐electrolysis of CO_2_ and H_2_O can render at the same time CO and H_2_, this is, a syngas equivalent (Lu *et al*., 2020). Syngas can also be catalytically transformed into methanol suitable for methylotrophs.

Finally, enzyme based electro‐catalysed production of HCOOH from CO_2_ has received great attention (Srikanth *et al*., 2014, 2017; Zhang *et al*., 2016; Schlager *et al*., 2017; Jayathilake *et al*., 2019). Effective oxygen tolerant biocatalysts capable of utilizing electrons supplied from a cathode are being sought to render biocatalytic HCOOH production from CO_2_ feasible. Bioelectrochemical CO_2_ reduction with enzymes or whole‐cell biocatalysts is generally characterized by a high selectivity of products and a high energy efficiency with a small overpotential to drive the desired reaction.

## Future prospects

The utilization of C1 raw materials is crucial for establishing a sustainable circular carbon economy. C1 compounds are envisioned as ideal resources for both the chemical industry and the biotechnological sector. Probably, the truly sustainable feedstock for a circular carbon economy is CO_2_ not only because its conversion to chemicals and fuels represents a sustainable solution for reducing greenhouse gas emissions, but also because it is abundant and can be obtained from different sources. Although direct CO_2_ capture from air will result in a net removal from the atmosphere, this process possesses technical and economic problems because it is highly dilute, only about 400 ppm, i.e. 100–300 times more dilute than in gas‐ and coal‐fired power plants. The estimated cost of capturing CO_2_ from air ranged from $300 to $1000 per ton. Thus, alternatively, the industrial production of chemicals from CO_2_ should consider the use of CO_2_ high‐volume waste as raw material (Bui *et al*., 2018).

Solar energy is envisioned as the most suitable renewable energy source to reduce CO_2_ and provide a sustainable system. Besides the firstly discovered Calvin–Benson–Bassham (CBB) cycle, other five natural CO_2_ fixation pathways have been described, i.e. the Wood‐Ljungdahl pathway, the reductive TCA cycle, the dicarboxylate/4‐hydroxybutyrate cycle, the 3‐hydroxypropionate bicycle and the 3‐hydroxypropionate/4‐hydroxybutyrate cycle. Refining the efficiencies of the native pathways as well as the design of synthetic pathways will provide new opportunities to improve the assimilation efficiencies of CO_2_. Developing new artificial autotrophic microorganisms, and especially phototrophic ones, for reinforcing carbon capture utilization (CCU) should be consider a key target in the next years. However, the use of artificial autotrophic cell factories still requires additional improvements in the CO_2_ fixation pathways, in order to solve compartmentalization, and to decide the best host as well as to reduce the cost of power supply. The increasing number of genomic and metagenomic sequences can help in this task, since it will allow finding by data mining better enzymes and pathways to improve the efficiency. In the same way, solar‐powered electrochemical reduction in CO_2_ and H_2_O to syngas, coupled to bacterial fermentation, can be also considered as an alternative to the sustainable production of useful chemicals (Haas *et al*., 2018).

Although it has been demonstrated that syngas, CO or CO_2_ can be directly transformed at industrial scale by acetogenic fermentation in useful alcohols (e.g. ethanol, butanediol), the main product generated by acetogenic bacteria is acetate. The potential of acetate to become a next‐generation platform substrate for its further fermentation into value‐added bioproducts has been underexplored so far (Kiefer *et al*., 2021; Kim *et al*., 2021).

Attractive platforms involving photomixotrophic metabolism in cyanobacteria can provide unparalleled improvements in yield for the conversion of CO_2_. Of particular interest is the ability to combine CO_2_ with other C1 compounds such as CH_4_ or chemically produced CH_3_OH and HCOOH (Kanno *et al*., 2017; Singh *et al*., 2018).

The creation of artificial bacterial consortia to improve the efficiency of C1 conversion into chemicals is a promising alternative (Hays *et al*., 2017). Strategies involving co‐cultivation of methanotrophic and oxygenic photosynthetic bacteria in biogas have been already explored (Van der Ha *et al*., 2012; Hill *et al*., 2017). An engineered *Synechococcus elongatus* able to convert CO_2_ into secreted sucrose can be used in co‐culture with other bacteria to generate biotechnological applications (Löwe *et al*., 2017; Weiss *et al*., 2017; Fedeson *et al*., 2020; Zhang *et al*., 2020b).

A proof‐of‐concept experiment conducted by Cheng *et al*. (2009) demonstrated that a biocathode enriched with the methanogenic archaea *Methanobacterium palustre* can store electricity in the form of CH_4_. In this CH_4_‐producing bioelectrochemical system (BES), CO_2_ and electrical energy are converted into CH_4_, using electrodes that supply either electrons or H_2_ to the archaea (Blasco‐Gómez *et al*., 2017). This technology is referred to as bioelectrochemical power‐to‐gas (BEP2G) and considered as a way of storing renewable surplus electricity (Geppert *et al*., 2016), i.e. CH_4_ generated with excess renewable power that cannot be fed into the electric grid can be directly stored in the existing gas infrastructures.

The main objective of the so call third‐generation‐(3G)‐biorefineries is to use cell factories to convert renewable energies and CO_2_ into chemicals, searching for routes for biomanufacturing chemicals in a carbon‐neutral manner. However, there are still many trends and key challenges for future advancement to make them competitive with the petroleum industry (Liu *et al*., 2020). Within this challenge, the design of efficient CO_2_ reduction systems by mimicking the mechanism of natural photosynthesis using semiconducting nanomaterials interfaced with electroactive bacteria in a photosynthetic microbial electrosynthesis system opens a revolutionary alternative (Xu *et al*., 2020; Gupta *et al*., 2021).

Finally, although the opportunities offered by the bioeconomy linked to the use of C1 compounds are wide and very promising, today there are still few companies that have started or are exploring the implementation of these biotechnological processes to industrial scale (Teixeira *et al*., 2018). Table 1 tries to summarize some examples of the main industrial approaches without pretending to be exhaustive. While CH_3_OH was explored years ago at industrial scale to produce single cell protein by bacteria or yeasts, the C1 liquids, neither CH_3_OH nor HCOOH are currently being used at industrial scale as feedstock to produce materials of commercial interest through fermentation. Interestingly, Feedstocks United (Netherlands) has developed a new technology that uses trioxane derived by chemical synthesis from C1 compounds as feedstock for microbial fermentation, exemplifying that there are still other options to be explored in the field of C1 biotechnology. All this leads to the conclusion that we are facing a large scenario of opportunities and strategies for biotechnological companies to face the challenge posed by the Green Deal in the coming years.

**Table 1:** List of companies that use C1 compounds as raw materials for microbial fermentation. Some industrial alliances are shown in parentheses.

C1 compound	Company	Final product
Syngas	LanzaTech (Basf, Global Bioenergies, Evonik, ArcelorMittal, Aemetis, IndianOil, Swayana)	Ethanol, butanediol, chemicals
Syngas	Ineos Bio (New Planet Bioenergy)	Ethanol
Syngas	Coskata (Synata Bio)	Ethanol
CH_4_	Newlight Technologies	Polyhydroxyalkanoates
CH_4_	Mango Materials	Polyhydroxyalkanoates
CH_4_	Calysta (BP, Cargill, NouriTech)	Protein, chemicals
CH_4_	Unibio	Protein
CH_4_	Industrial Microbes	Methanol
CH_4_	MBP Titan (formerly Intrexon)	Protein, chemicals
CH_4_	NatureWorks (Calysta)	Lactic acid
CO_2_	Deep Branch	Protein
CO_2_	Solar Foods	Protein
CO_2_	Air Protein	Protein
CO_2_	Novo Nutrients	Protein
CO_2_	Kiverdi	Protein
CO_2_	White Dog Labs	Protein
CO_2_	OPX Biotechnologies (Cargill)	Biofuels
CO_2_	Trelys	Amino acids
CO_2_	BioMason (Novo Holdings)	Biocementation
CO_2_	BioCement Technologies Inc.	Biocementation
CO_2_	Basilisk	Self‐healing concrete
HCOOH‐electro CO_2_	Ginkgo Bioworks	Biofuels

## Conflict of interest

None declared.
